# Induction of liver transplant immune tolerance in an outbred rat strain model using tacrolimus

**DOI:** 10.1186/s42826-023-00156-5

**Published:** 2023-03-08

**Authors:** Min-Jung Park, Hyun Sik Na, Young-Shin Joo, Keun-Hyung Cho, Se-Young Kim, Jeong Won Choi, Jin-Ah Baek, Jong Young Choi, Young Kyoung You, Mi-La Cho

**Affiliations:** 1 The Rheumatism Research Center, Catholic Research Institute of Medical Science, College of Medicine, The Catholic University of Korea, Seoul, 06591 Republic of Korea; 2 Lab of Translational ImmunoMedicine, Catholic Research Institute of Medical Science, College of Medicine, The Catholic University of Korea, Seoul, Republic of Korea; 3 Department of Biomedicine & Health Sciences, College of Medicine, The Catholic University of Korea, 222, Banpo-daero, Seocho-gu, Seoul, 06591 Republic of Korea; 4 Department of Laboratory Animal Research Center, Catholic Medical Center, Institute of Biomedical Industry, The Catholic University of Korea, Banpo-daero, Seocho-gu, Seoul, Republic of Korea; 5 Division of Hepatology, Department of Internal Medicine, Seoul St. Mary’s Hospital, College of Medicine, The Catholic University of Korea, Banpo-daero, Seocho-gu, Seoul, Republic of Korea; 6 Department of Surgery, Seoul St. Mary’s Hospital, College of Medicine, The Catholic University of Korea, Banpo-daero, Seocho-gu, Seoul, Republic of Korea; 7 Department of Medical Life Sciences, College of Medicine, The Catholic University of Korea, 222, Banpo-daero, Seocho-gu, Seoul, 06591 Republic of Korea; 8 Impact Biotech, Seoul, 137-040 Republic of Korea

**Keywords:** Tacrolimus, Orthotopic liver transplantation, Rejection, Inflammatory cytokine, Th1 cell, Th17 cell, Immune tolerance

## Abstract

**Background:**

Orthotopic liver transplantation is the only option for patients with end-stage liver disease and hepatocellular carcinoma. Post-transplant immunosuppressive therapy is important to prevent graft failure. We investigated the effectiveness of tacrolimus (FK506) and their mechanisms for liver transplant immune tolerance in an outbred rat LT model.

**Results:**

To investigate the therapeutic effect of the FK506 on outbred rat LT model, FK506 and postoperative therapy were administered subcutaneously once or twice daily to transplanted rats. Histopathological and immunohistochemical analyses were conducted for all groups. The regulation of inflammatory cytokine signaling in the spleen was analyzed by flow cytometry. FK506 attenuated allograft rejection and increased survival in rat orthotopic liver transplantation models. The FK506-treated group had reduced serum levels of alanine aminotransferase, aspartate aminotransferase and alkaline phosphatase. Furthermore, FK506 decreased the expression of inflammatory cytokines and the activation of pathogenic Th1 and Th17 cells in the liver.

**Conclusions:**

Taken together, we revealed that FK506 ameliorated strong allograft rejection in outbred liver transplantation model by anti-inflammatory effect and inhibitory peroperty of pathogenic T cells.

## Background

As the number of patients with liver disease increases, the frequency of liver transplantation (LT), which is the final treatment method, is increasing. LT is the treatment of choice for end-stage liver disease and acute liver failure, based on overall survival and quality of life [1, 2]. The success of LT and long-term survival depend on the use of immunosuppressant drugs. However, the long-term (lifetime) use of immunosuppressive drugs after transplantation has serious side effects, such as cancer, infection, inflammation, and destruction of the immune-defense system [3, 4]. Thus, a major goal in transplantation is to induce immunological self-tolerance and homeostasis.

LT is a complex procedure that involves the safe extraction and preparation of the donor liver, removal of the recipient's diseased liver, and then anastomosis of the blood vessels and biliary tract [5, 6]. In rats, the most common technique is orthotopic liver transplantation (OLT), in which the native liver is removed and replaced by the donor organ in the same anatomic position [7, 8]. However, rat transplantation surgery requires a long training period to achieve a high success rate using the fast, accurate, and delicate surgical procedures required for small animals. The rat OLT model produces more clinically relevant and reliable data than does the mouse OLT model [6].

In animal LT, liver allografts are commonly accepted across fully mismatched major histocompatibility complexes (MHCs) without immunosuppression [9]. In inbred mice, liver grafts in many fully allogeneic combinations survive permanently without the administration of immunosuppressants [10]. Inbreeding depression and the lack of genetic diversity in inbred mice, however, could mask unappreciated causes of graft failure or remove barriers to tolerance induction. Human genes are not identical. In this study, we used outbred rats with genetic diversity in donors and the recipients to mimic the clinical situation.

Tacrolimus (FK506) has become the first-line immunosuppressant drug used after LT [11–13]. We determined the therapeutic efficacy of FK506 in an outbred rat LT model. This model, which features a strong immune response, contributed to our understanding of the mechanisms underlying the induction of liver transplant immune tolerance by FK506. The aim of this study was to determine whether FK506 could mitigate allograft rejection and to clarify their mechanisms underlying the induction of liver transplant immune tolerance in an outbred rat LT model.

## Results

### Post-transplantation FK506 treatment improved graft failure in an outbred liver transplantation model

**Fig. 1:**
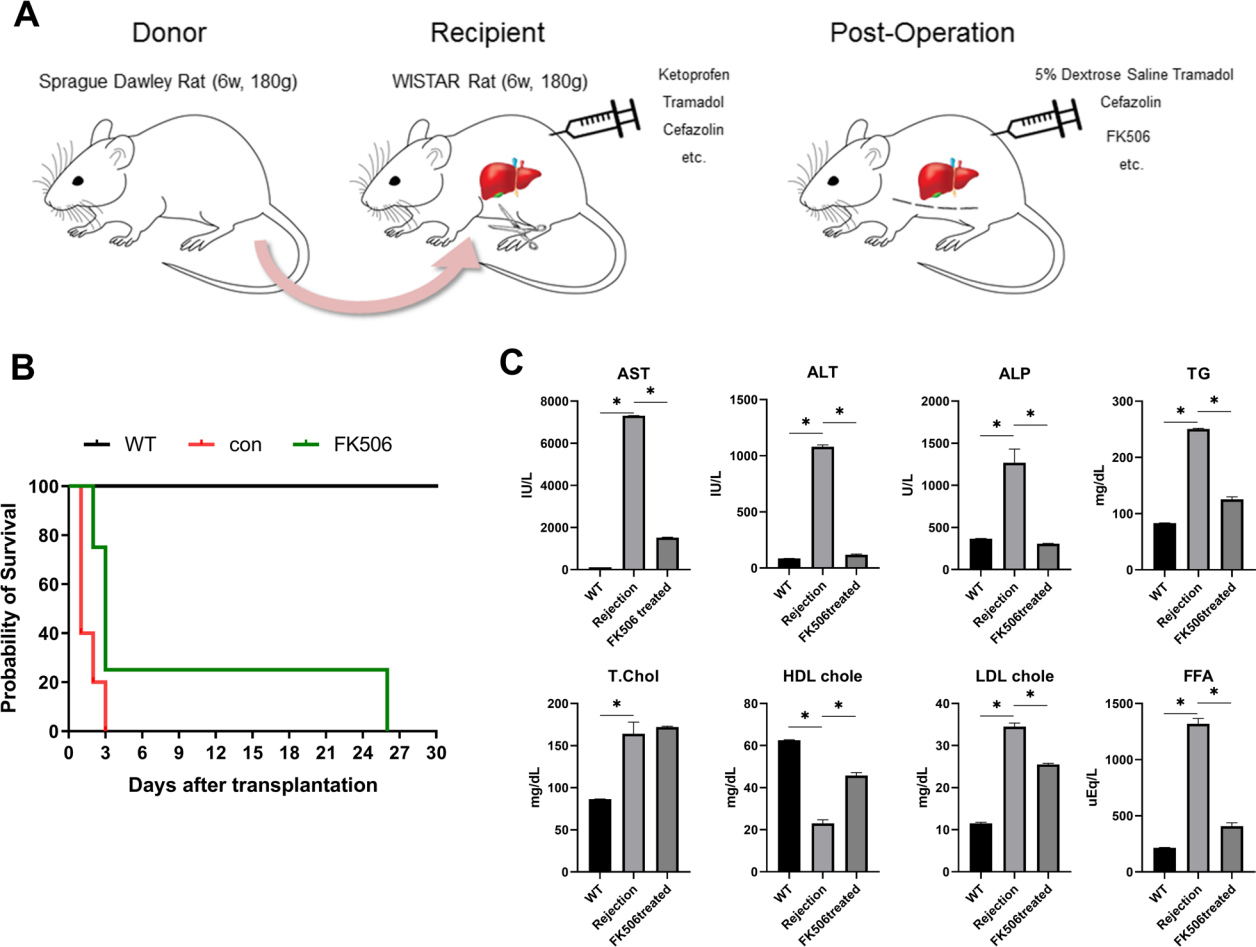
Tacrolimus attenuates liver allograft rejection in rats. **A** Orthotopic liver transplantation was performed in SD and Wistar rats. Postoperative therapy was administered daily until sacrifice. **B** Survival rates in recipients (control and 1 mg/kg FK506–treated groups). **C** Changes in serum levels of AST, ALT, ALP, TG, total cholesterol, HDL cholesterol, LDL cholesterol, and FFAs in all groups at day 3 after LT. The data are reported as the means ± SDs from three independent experiments. *P < 0.05

In previous studies, OLT was performed using inbred rats; in this study, it was performed in outbred rats who were treated with FK506 to increase survival and reduce organ rejection (Fig. 1A, B). Drug therapy was administered before and after surgery to increase the operation success rate. The treatment given to liver-transplant recipient rats included antibiotics, painkillers, and FK506 (Table 1). Metabolic index assays were conducted to measure liver function. The FK506-treated group showed reduced serum levels of alanine aminotransferase (ALT), aspartate aminotransferase (AST) and alkaline phosphatase (ALP) and triglyceride (TG) compared to rejection group. Moreover, the FK506-treated recipient rats showed reduced levels of LDL cholesterol and FFAs, and increased levels of HDL cholesterol (Fig. 1C).

**Table 1:** Pre-and post- management for liver transplantation

Animals		Treatments			
Donor	Recipient	Pre-operation		Post-operation	
Sprague Dawley Rat (6w, 180 g)	WISTAR rat (6w, 180 g)	Drug	Dosage	Drug	Dosage
		Atropine	0.05 mg/kg, SC	0.9% Saline	3 mL, Twice a day, SC
		Ketoprofen	5 mg/kg, SC	Cefazolin	30 mg/kg, once a day, SC
		Tramadol	5 mg/kg, SC	Ketoprofen	5 mg/kg, Twice a day, SC
		Cefazolin	30 mg/kg, SC	Tramadol	5 mg/kg, once a day, SC
		Dexamethasone	5 mg/kg, SC	Ranitidin	5 mg/kg, Twice a day, SC
				FK506	1 mg/kg, Twice a day, SC

### FK506 attenuates pro-inflammatory response and fibrosis in an outbred LT model

**Fig. 2:**
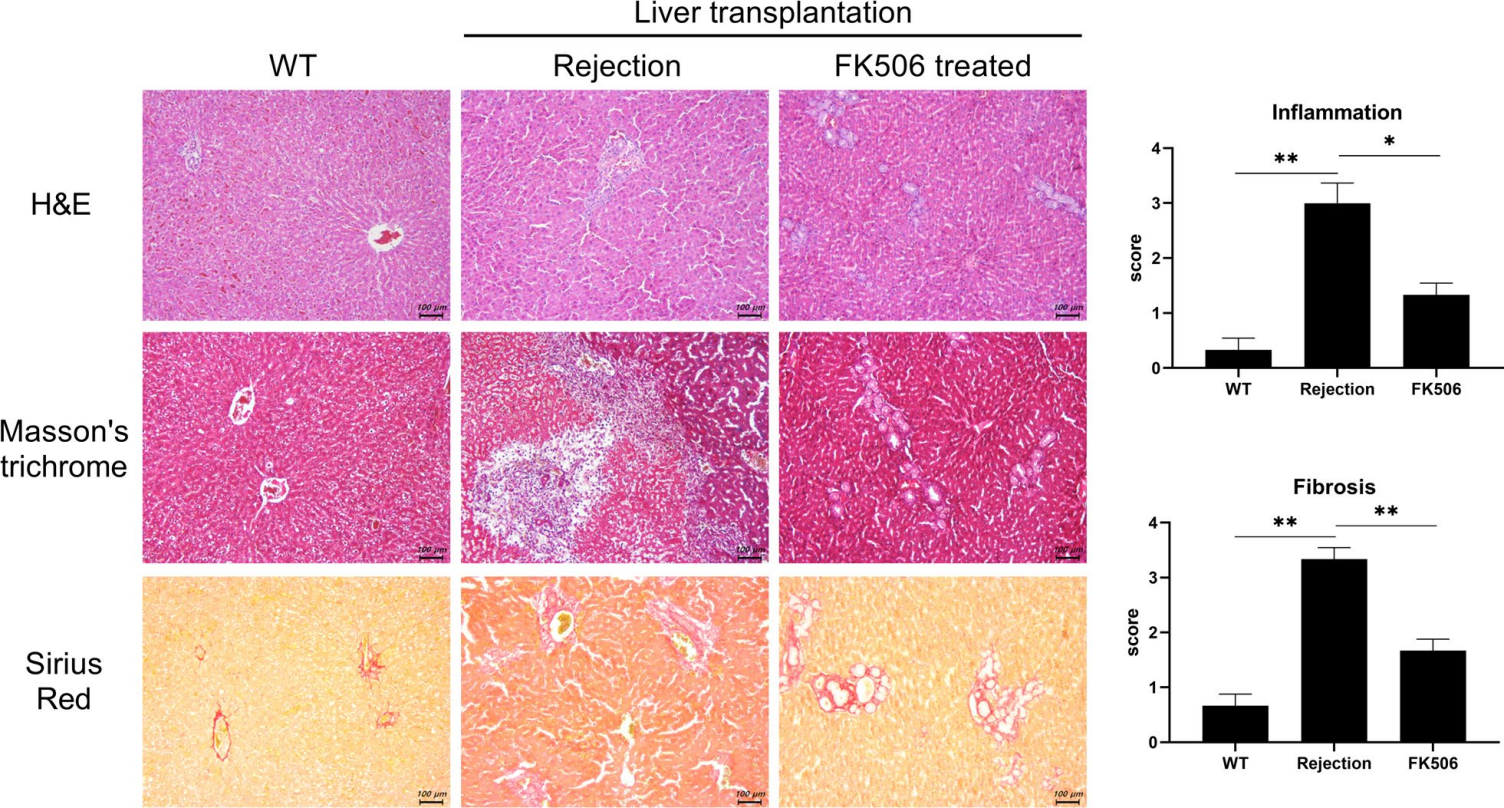
Tacrolimus alleviates liver graft injury and fibrosis. Morphological changes in grafts after LT. Liver tissues were acquired from the control and FK506 (1 mg/kg) groups at day 3 after LT and analyzed by staining with H&E, MT, and Sirius red to evaluate the severity of inflammation and fibrosis. The data are reported as means ± SDs from three independent experiments. *P < 0.05, **P < 0.01

**Fig. 3:**
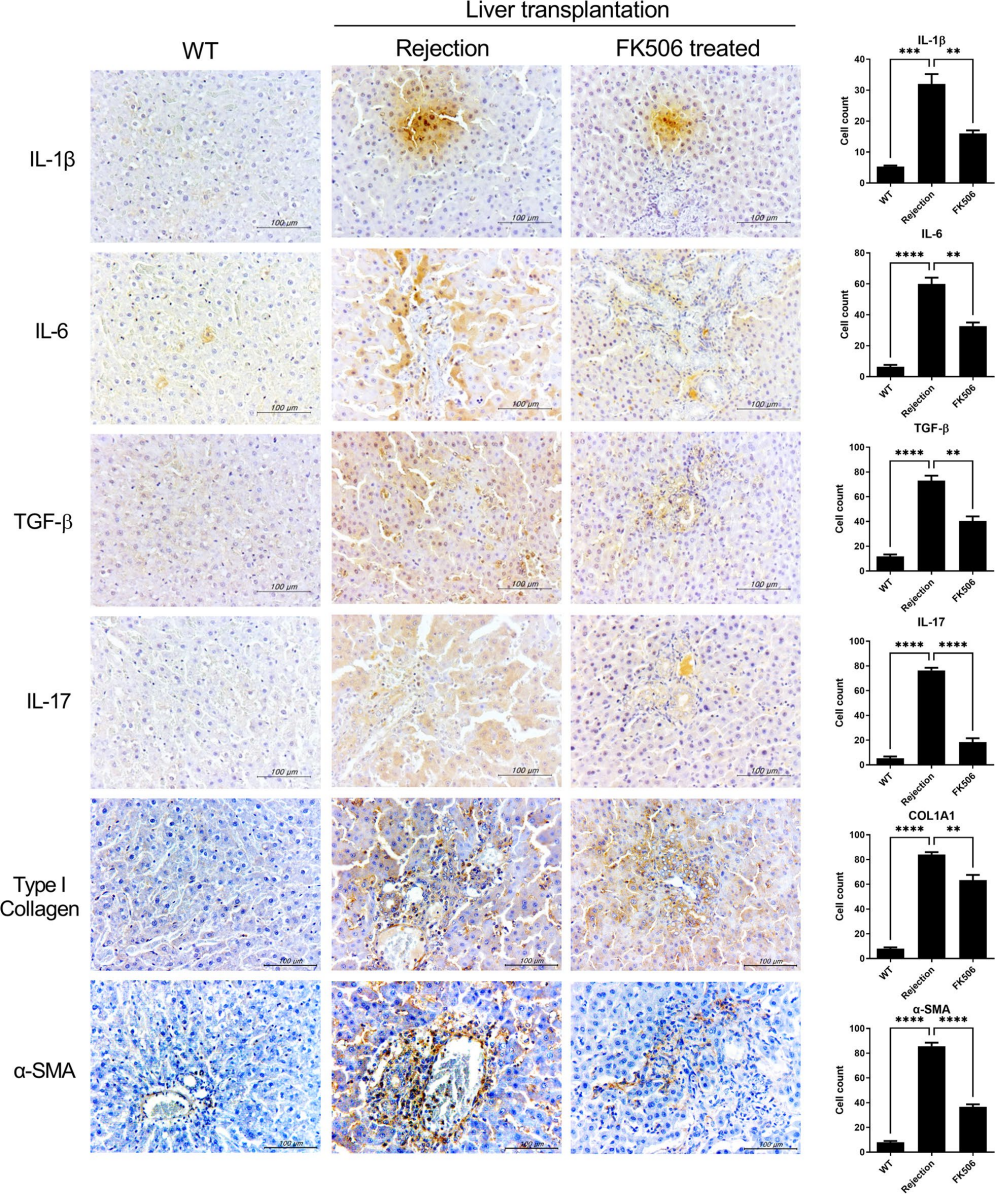
Tacrolimus decreases the expression of hepatic proinflammatory cytokines. The expression of IL-1β, IL-6, TGF-β, IL-17, Type I collagen and α-SMA in the transplanted livers was assessed by immunohistochemistry. Positive cells for each antibody are shown on the right. The data are reported as means ± SDs from three independent experiments. **P < 0.01, ***P < 0.005, ****P < 0.001

Fibrosis and infiltration of inflammatory cells were analyzed through hematoxylin and eosin (H&E) and Masson’s trichrome (MT), and Sirius red staining. Liver tissue from the rejection groups showed significantly severe liver damage with infiltration of inflammatory cells, collagen deposition, and hepatic fibrosis. The infiltration of inflammatory cells and fibrosis were reduced in liver tissue from the FK506-treated groups compared to rejection group (Fig. 2). Additionally, we used immunohistochemistry to detect inflammatory mediators and fibrosis markers in the transplanted livers. Administration of FK506 decreased the expression of interleukin (IL)-1β, IL-6, IL-17, and transforming growth factor beta (TGF-β) in liver tissues (Fig. 3). Also, the expression of fibrosis markers which were α-sma and Collagen I alpha 1(Col-1) were less infiltration in liver tissues from FK506 treated group compared to those from rejection group. Thus, FK506 ameliorated allograft rejection by downregulating the expression of inflammatory and fibrogenic factors.

### Tacrolimus suppressed in vivo effector T-cell responses in an outbred LT model

**Fig. 4:**
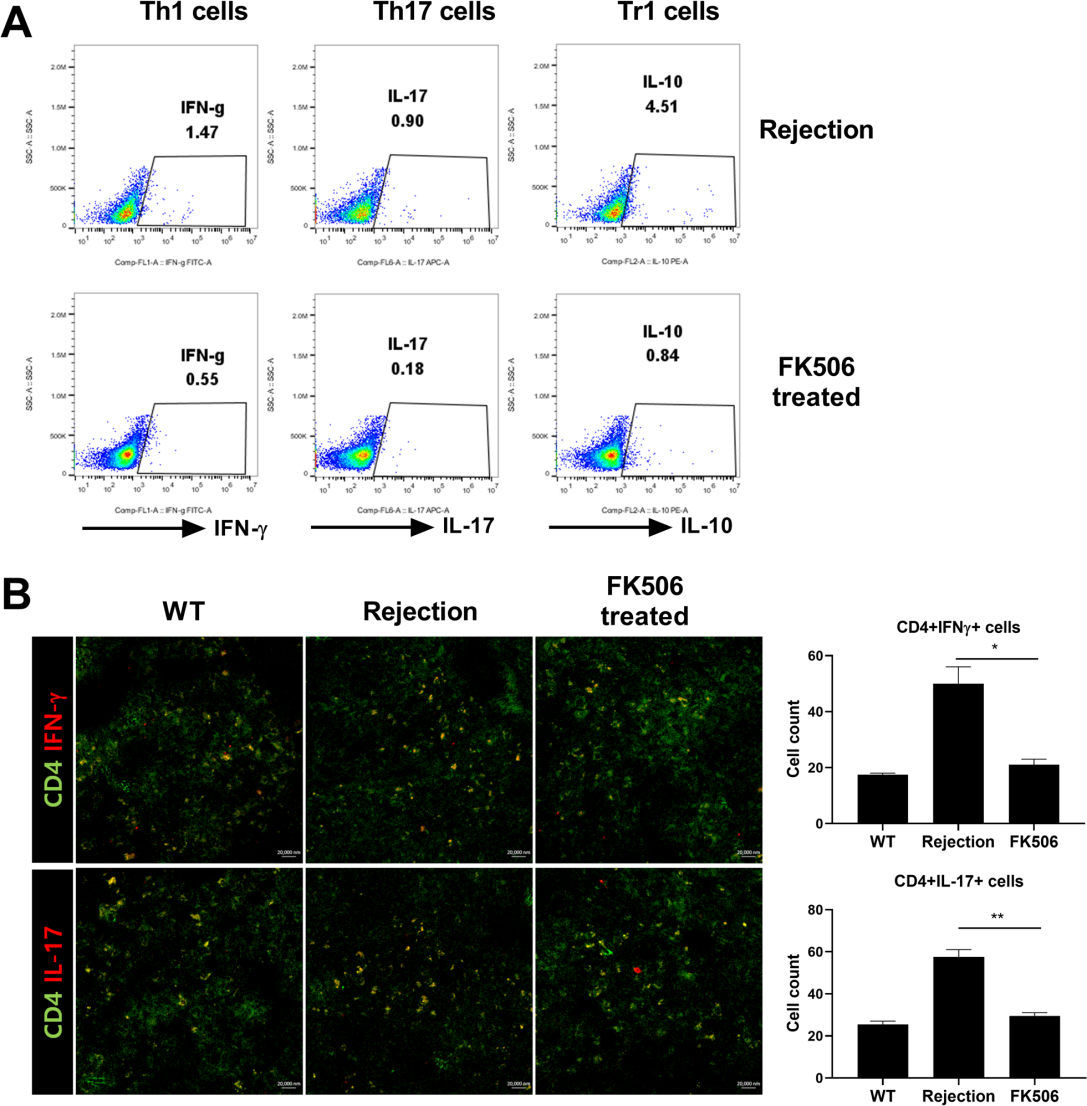
Tacrolimus inhibits Th1 and Th17 cell activation. **A** The expression of IFN-g, IL-17, and IL-10 in the spleen from all groups at day 3 after LT was assessed by flow cytometry with gating on CD4^+^IFN-γ^+^, CD4^+^IL-17^+^, or CD4^+^IL-10^+^ cells. Data are representative of at least two independent experiments. **B** Spleens were examined by immunofluorescence staining with monoclonal antibodies against CD4 (green) and IL-17 (red). *P < 0.05, **P < 0.01. Original magnification, × 400

To investigate the in vivo mechanism underlying the action of FK506 in the outbred LT model, we counted the number of CD4^+^ interferon (IFN)-γ^+^, CD4^+^IL-17^+^, and CD4^+^IL-10^+^cells in splenocytes isolated from each group using flow cytometry. The numbers of CD4^+^IFN-γ^+^ and CD4^+^IL-17^+^ T cells in spleens from FK506-treated LT model rats were decreased compared with those in the rejection group. The numbers of CD4^+^IL-10^+^cells in the splenocytes were also reduced in the FK506-treated LT model compared with those in the rejection group (Fig. 4A). Furthermore, confocal microscopic analysis showed decreases in the numbers of CD4 + IFN-γ + and CD4 + IL-17 + T cells in the spleens of FK506-treated rats (Fig. 4B). These results suggest that the alleviation of acute allograft rejection following transplantation with FK506-treated splenocytes is the result of the suppression of the pathogenic T-cell response.

## Discussion

In the inbred LT model, liver allografts can be accepted across mismatched MHCs without immunosuppressive drug therapy [14]. Unlike in the outbred system, the rate of successful transplant engraftment is high in inbred mouse models. However, human systems are very different from inbred mouse systems. The human outbred population contains an excess of heterozygotes. The genetic diversity and heterosis in the outbred human population have been suggested to make successful organ transplantation very difficult [15, 16]. To establish the outbred rat LT model in this study, we used outbred Wistar and SD rats, which are typically used in transplantation and functional tests. We created a strong rejection model using outbred rats to approximate the clinical situation. This model will deepen our understanding of the mechanisms underlying LT tolerance.

Immunosuppressive drugs are commonly used following LT [11]. Calcineurin inhibitors, including FK506, serve to prevent transcription of the autocrine factor IL-2, preventing cell proliferation [17]. FK506 greatly reduced the rate of allograft rejection, although the chronic use of such drugs is marred by a range of side effects, including vascular toxicity [13]. We found that FK506 treatment significantly prolonged graft survival and reduced hepatic inflammation. In addition, FK506 suppressed pro-inflammatory T helper (Th)1/Th17 responses [18, 19] and Foxp3 + Treg [20]. However, FK506 also inhibits immunomodulatory IL-10 cytokines [21]. In a mouse transplantation model, the genetic delivery of IL-10 to allografts led to prolonged graft survival [22, 23]. Novel immunomodulatory regimens that maximize beneficial effects for transplantation tolerance without the aforementioned adverse effects are needed. Further research is needed to determine the minimum effective FK506 dosage when used in combination with immunoregulatory drugs.

Blood chemistry tests showed that liver function was improved [24]. The levels of enzyme-related liver metabolites (ALT, AST, and ALP) were significantly reduced in the group treated with FK506 compared with the rejection group. The ALT and ALP levels were restored to similar levels as in the wild-type (WT) group.

Interestingly, FK506 treatment also had significant effects on lipid metabolism. The TG level was lower than in the rejection group and similar to that in the WT group. The total cholesterol level was slightly higher than in the rejection group, but the levels of low-density lipoprotein (LDL) cholesterol and free fatty acid (FFA) were low; the high-density lipoprotein (HDL) cholesterol level was higher and close to that in the WT group. Considering that fatty liver disease is a common factor for LT and is likely to recur after transplantation, FK506 represents a new alternative post-LT treatment for patients with fatty liver disease.

## Conclusions

In this study, we found that an outbred hepatic transplantation model led to robust immune rejection and that FK506 induced long-term allograft acceptance.

## Methods

### Animals

Six-week-old male Wistar and Sprague Dawley (SD) rats (male, 180–220 g at baseline) were purchased from Japan SLC Inc. (Shizuoka, Japan) by Central Lab Animal Inc. (Seoul, South Korea). A maximum of three animals per cage were housed in a room with controlled temperature (20–26 °C) and light (12/12-h light/dark cycle) conditions. The rats had free access to a gamma ray–sterilized diet (TD 2018S; Harlan Laboratories Inc., IN, USA) and autoclaved R/O water. All animal experiments were performed in accordance with the Animal Care and Use Committee of the Catholic University of Korea (approval no. CUMS-IACUC-2017-0215-03). All procedures performed followed the ethical guidelines on animal use.

### Orthotopic liver transplantation

The animals were divided into three groups: a non-surgical control group and surgical groups with and without FK506 treatment (n = 5/group). LT recipients were premedicated with atropine sulfate [0.05 mg/kg, subcutaneous (SC) injection; JE IL Pharm. Co., Ltd., Korea], heparinized saline (100 ml/kg, intravenous injection; heparin 5000 IU/ml, JW Pharm. Co., Korea; 0.9% normal saline, Dai Han Pharm. Co. Ltd., Korea), ketoprofen (5 mg/kg, SC, 100-mg ketoprofen injection; SDC Pharm. Co., Korea), tramadol (5 mg/kg, SC, 50-mg tridol injection; YUHAN Co., Korea), and cefazolin (30 mg/kg, SC, 1-mg cefazolin injection; Chong Kun Dang Pharmaceutical Corp., Korea). Following surgery, normal saline (Dai Han Pharm. Co. Ltd.) or 5% dextrose saline (5% dextrose and sodium chloride injection, Dai Han Pharm. Co. Ltd.) was administered subcutaneously. Dexamethasone (5-mg/kg cortisol injection, SC; HanAll Biopharma, Seoul, S. Korea) was administered once during surgery, at the moment of reperfusion. Anesthesia was carried out using an anesthetic machine (Harvard, USA) and isoflurane (2–3.5%, ifran; Hana Pharm. Co. Ltd., Seoul, S. Korea) during the LT operation. All procedures performed in this study were OLTs based on a modified version of Kamada’s technique under a × 5-magnified surgical field using a surgical loupe [5, 8, 25]. The donor rat was placed in the supine position. Long midline and additional right transverse incisions were made to ensure adequate exposure. After inspection for the presence of perihepatic anomaly, the phrenic vein was ligated and divided using 7–0 silk sutures. All suspensory ligaments surrounding the liver were divided. The bile duct was dissected and transected in the supraduodenal area with enough length remaining after cuff insertion (24-gauge angiocatheter; Terumo, Tokyo, Japan). The retroperitoneal connective tissues were dissected, the right adrenal vein was ligated, and the infrahepatic inferior vena cava (IVC) was freed. The portal vein was prepared for perfusion, and the pyloric vein was ligated and divided to enable portal vein cuff manipulation. Simultaneous cross-clamping of the infrahepatic IVC and the portal vein was performed. The graft was perfused through the portal vein with minimal pressure using ~ 10 ml cold heparinized saline, and venting was performed through the right atrium using a transdiaphragmatic approach. The liver graft was retrieved from the cold-preservation saline solution, and bench surgery was performed in preserved saline solution. The attached diaphragmatic tissue was dissected, and the portal vein cuff (polyethylene tube, 6 FG, outside diameter 2.1 mm; Havard Apparatus, USA) was inserted. The total preservation time, including the bench procedure, was approximately 90 min. Immediately following the donor operation, a total hepatectomy was performed in the recipient in almost the same manner as the donor surgery. The main difference was that the transections were made close to the liver to secure sufficient lengths of the bile duct, portal vein, and IVC to perform the reconstruction. Following total hepatectomy, the preserved graft liver was implanted. The suprahepatic IVC was reconstructed using 8–0 monofilament nylon continuous sutures. The infrahepatic IVC was reconstructed in the same end-to-end manner using 8–0 monofilament nylon continuous sutures. Following portal vein reconstruction, the suprahepatic, infrahepatic, and portal cross-clamps were released to restore the systemic and hepatic circulation. At this point, 2 ml saline and dexamethasone were injected subcutaneously. The total vascular clamping time was 20–25 min. Following bile duct reconstruction using the cuff technique, the abdominal wound was closed using 5–0 monofilament nylon sutures. All surgical procedures were performed under aseptic conditions. After surgery, the recipients were observed closely in warmed cages for ~ 1 h.

Postoperative medications were administered and included analgesics (ketoprofen, 5 mg/kg, SC, twice a day; tramadol, 5 mg/kg, SC, once a day), antibiotics (cefazoline, 30 mg/kg, SC, once a day), a histamine H2 receptor blocker (ranitidine HCl, 5 mg/kg, twice a day; Alcon, Korea), and FK506 (1 mg/kg, SC, twice a day). For the fluid therapy, normal saline or 5% dextrose saline was given, depending on the recipient’s condition. To help feed the animal, the animals were supplied a gel-type recovery diet (Nutra-Gel, F5769-Kit; Bio Serv, USA).

### Clinical evaluation and examination after orthotopic liver transplantations

Animals were randomly assigned to different experiments to evaluate the in vivo effects of FK506 on survival, serum parameter and histological analysis and T cell subset analysis. All animals of each groups (n = 3) were monitored until death to determine survival time.

### Biochemical analysis

ALT, AST, ALP, TG, LDL cholesterol, HDL cholesterol, and FFA levels were measured using commercial kits (Asan Pharmaceutical Co., Hwangseong-gi, Gyeonggi-do, Republic of Korea).

### Histopathological analysis

All liver tissues were collected from each group. The tissues were fixed in 10% formalin and embedded in paraffin. Sections of 4–5 μm thickness were cut, dewaxed using xylene, dehydrated through an alcohol gradient, and then stained with H&E and MT.

### Immunohistochemistry

Paraffin-embedded sections were incubated at 4℃ with the following primary monoclonal antibodies: anti–IL-1β, anti–IL-17, anti–IL-6, and anti–TGF-β. The samples were then incubated with the respective secondary biotinylated antibodies, followed by 30 min incubation with a streptavidin–peroxidase complex. The reaction product was developed using 3,3-diaminobenzidine chromogen (Dako, CA, USA).

### Flow cytometric analysis

Mouse lymphocytes were immunostained using fluorescently conjugated antibodies against CD4, IL-17, IL-10, and IL-4. For intracellular staining, cells were first stimulated for 4 h with phorbol myristate acetate (25 ng/mL) and ionomycin (250 ng/mL) in the presence of BD GolgiStop (BD Biosciences, San Jose, CA, USA). Intracellular staining was performed using a BD Cytofix/CytopermPlus Fixation/Permeabilization Kit and BD GolgiStop Kit (BD Biosciences). Flow cytometry was performed using a cytoFLEX Flow Cytometer (Beckman Coulter, Brea, CA, USA).

### Confocal staining

Spleen tissues were acquired from the liver-transplanted rats, snap frozen in liquid nitrogen, and stored at –80℃. Tissue cryosections (7 μm thick) were fixed in 4% paraformaldehyde and stained using FITC-conjugated anti-CD4, and PE-conjugated anti–IL-17 (eBioscience, San Diego, CA, USA). After incubation overnight at 4℃, the stained sections were analyzed using a confocal microscope (LSM 510 Meta; Zeiss, Gottingen, Germany). CD4 + IL-17 + T cells were enumerated visually at higher magnification (projected on a screen) by four individuals.

### Statistical analyses

Data are expressed as means ± standard errors of the mean. One-way analysis of variance with Bonferroni’s post-hoc test was used to compare data among three or more groups. Statistical significance was considered at P < 0.05. All statistical analyses were performed using Prism (standard version 8; GraphPad Software, San Diego, CA).

## Data Availability

All data are available in the manuscript or upon request to the authors.

## Abbreviations

ALT: Alanine aminotransferase
AST: Aspartate aminotransferase
IVC: Inferior vena cava
MHCs: Major histocompatibility complexes
MT: Masson’s trichrome
OLT: Orthotopic liver transplantation
